# Impact of Perioperative Antibiotic Prophylaxis in Caesarean Section on the Maternal Gut Microbiome: A Systematic Review

**DOI:** 10.3390/jcm14145104

**Published:** 2025-07-18

**Authors:** Elisabeth AL Feles, Claudio Neidhöfer, Christina Wessels, Rosalie Gruber, Frauke Mattner

**Affiliations:** 1 Division of Hygiene and Environmental Medicine, Department of Human Medicine, Faculty of Health, Witten/Herdecke University, Alfred-Herrhausen-Strasse 50, 58455 Witten, Germany; mattnerf@kliniken-koeln.de; 2 Institute of Hygiene, Cologne Merheim Medical Centre, University Hospital of Witten/Herdecke, Ostmerheimer Strasse 200, 51109 Cologne, Germany; wesselsc@kliniken-koeln.de (C.W.);; 3 Central Pharmacy, Cologne Merheim Medical Centre, University Hospital of Witten/Herdecke, Ostmerheimer Strasse 200, 51109 Cologne, Germany; 4 Division of Clinical Bacteriology and Mycology, University Hospital of Basel, Petersgraben 4, 4031 Basel, Switzerland; claudio.neidhoefer@usb.ch

**Keywords:** gut microbiome, maternal microbiome, caesarean section, perioperative antibiotic prophylaxis, microbial diversity, maternal–fetal interface, obstetric antibiotic exposure

## Abstract

**Background/Objectives:** Caesarean section (CS) accounts for over 20% of global births and routinely involves perioperative antibiotic prophylaxis (PAP) to reduce surgical site infections. While the impact of such prophylaxis on neonatal microbiome development is well described, effects on the maternal gut microbiome remain underexplored. This systematic review synthesizes current evidence on how antibiotic prophylaxis during CS affects maternal gut microbiome composition and diversity—an underrepresented, but clinically relevant aspect of maternal–fetal medicine. **Methods:** A systematic literature search was conducted in Medline (PubMed), the Cochrane Library, and the WHO International Clinical Trials Registry Platform (ICTRP) through November 2024. Inclusion criteria were defined according to the Preferred Reporting Items for Systematic Reviews and Meta-Analyses (PRISMA) guidelines. Eligible studies used molecular techniques to report maternal gut microbiome outcomes (alpha- and beta-diversity). The search concentrated on beta-lactam antibiotics. Reference lists were screened, but no additional grey literature was searched. Synthesis followed the Synthesis Without meta-analysis (SWiM) approach. No review protocol was registered. The review received no external funding. **Results:** Out of 1011 records, three studies (total 286 mothers) met the inclusion criteria. All reported maternal microbiome outcomes secondarily to infant-focused research. Only one study provided pre- and post-birth stool samples. Applied antibiotic regimens, sequencing methods, and reported microbiome metrics for alpha- and beta-diversity varied considerably, thus limiting comparability of results. Due to high heterogeneity, no formal risk of bias was assessed. While taxonomic diversity changes were inconsistent, significant shifts in functional diversity metrics were observed postpartum. **Conclusions:** Evidence on maternal microbiome disruption following perioperative antibiotic prophylaxis in CS is methodologically fragmented and limited by small sample sizes and inconsistent antibiotic protocols. Nonetheless, functional diversity appears sensitive to antibiotic exposure. To improve clinical understanding and safety, maternal-focused studies using standardized protocols are urgently needed. The maternal microbiome may play a key role in both recovery and shaping the newborn’s early microbial environment.

## 1. Introduction

Caesarean section (CS) rates have increased markedly in recent decades, accounting for over 20% of global births [1]. In high-income countries, rates typically range between 25% and 35%, while some regions, such as Latin America and the Caribbean, report rates exceeding 40%—the highest worldwide [2].

While CS can be life-saving for both mother and child in selected indications, it is associated with a substantially higher risk of surgical site infections (SSIs) compared to vaginal delivery (VD). In fact, CS has the third highest rate of SSIs among all surgical procedures [3]. To reduce this risk, perioperative antibiotic prophylaxis (PAP) is recommended by the World Health Organization (WHO) and widely adopted by national guidelines, including those in Germany, the UK, and the US [3,4,5,6]. Recommended agents include penicillins (e.g., ampicillin) and first- or second-generation cephalosporins (e.g., cefazolin or cefuroxime).

The efficacy of PAP in reducing maternal infectious morbidity is well established and supported by high-quality meta-analyses [7]. As knowledge about the role of the microbiome in maternal and neonatal health has expanded, interest has grown in understanding how obstetric interventions may influence microbial composition and function. This has also shed light on the interplay between maternal and neonatal microbial ecosystems.

Recent research has primarily focused on neonates, whose early microbial colonization is sensitive to both delivery mode and maternal antibiotic exposure. Numerous studies have demonstrated that perinatal antibiotics, as used in CS, are associated with altered microbial succession in neonates, including reduced Bifidobacteria abundance, delayed colonization, and increased hospital-associated taxa [8,9].

In contrast, little is known about the effects of PAP on the maternal gut microbiome. Pregnancy itself is associated with a physiological decline in microbial alpha-diversity [10,11] and increased metabolic load, potentially reducing microbial resilience [12]. The combination of hormonal modulation, immunological shifts, and surgical stress may amplify microbiome vulnerability at the time of delivery [13,14]. Despite its potential relevance for maternal recovery, immune function, and long-term health, the maternal microbiome remains a largely overlooked dimension of perinatal research.

This systematic review synthesizes current evidence on how PAP during CS affects maternal gut microbiome composition and diversity. It examines both taxonomic and functional changes, highlights methodological limitations, and identifies gaps that hinder clinical interpretation. Understanding these dynamics is essential for a more comprehensive view of maternal–fetal health, particularly in the context of rising CS rates and expanding antibiotic use in obstetric care.

## 2. Materials and Methods

This systematic review was conducted in accordance with the Preferred Reporting Items for Systematic Reviews and Meta-Analyses (PRISMA) 2020 guidelines [15] and the PRISMA-S extension [16]. Both checklists are provided in the Supplementary Files S4 and S5. No formal review protocol was registered (e.g., in PROSPERO). Nevertheless, the review was conducted according to the above-mentioned guidelines without any amendments to the search strategy or eligibility criteria.

Due to the heterogeneity of study designs and outcome measures, results were synthesized following the Synthesis Without Meta-analysis (SWiM) approach [17].

A comprehensive literature search was carried out in three databases: Medline (via PubMed), the Cochrane Library, and the World Health Organization International Clinical Trials Registry Platform (ICTRP). The search covered all records from database inception to November 2024, without restrictions on publication language or status. The full search strategy is available in Supplementary File S1. In addition, the reference lists of all full-text articles screened were manually checked for potentially relevant studies. No further browsing of grey literature sources or print proceedings was conducted. However, the ICTRP search retrieved one ongoing study by the authors (“MAMA Study”, DRKS00027305) that was still listed as ‘recruiting’ at the time of the search and did not provide published results. Therefore, this record was not eligible for inclusion in the final synthesis.

The initial search was constrained to studies investigating the use of cefuroxime, as this is the only agent explicitly approved in Germany for perioperative prophylaxis during CS [18]. To increase sensitivity and account for international practice, the search was subsequently expanded to include all beta-lactam antibiotics.

The search strategy was developed in accordance with the Cochrane Methodological Expectations of Cochrane Intervention Reviews (MECIR) Manual and utilized the PICO framework with the following parameters [19]:

- Population (P): mothers undergoing Caesarean section,
- Intervention (I): perioperative antibiotic prophylaxis with cefuroxime or other beta-lactams,
- Comparison (C): no antibiotic intervention or alternative timing/regimen,
- Outcome (O): changes in maternal gut microbiome, specifically alpha- or beta-diversity metrics assessed by molecular techniques (e.g., 16S rRNA or shotgun sequencing).

Only original studies using culture-independent microbiome analysis were included. Reviews, meta-analyses, and studies relying solely on culture-based methods were excluded.

To ensure reproducibility, screening and selection were performed independently by two reviewers (EF and RG), with discrepancies resolved by consensus. Full-text assessment and data extraction were carried out by EF in consultation with CW. Identified studies were assessed for relevance and methodological quality.

Assessing risk of bias is a crucial component of systematic reviews because it determines the confidence that can be placed in the included evidence. In this review, however, the number of eligible studies was very small, the study populations were limited in size, and the methodological approaches and reported outcomes were highly heterogeneous. Moreover, all included studies were primarily observational and did not focus on the maternal microbiome as a primary outcome. Standard risk of bias tools such as the Newcastle–Ottawa Scale for cohort studies or ROBINS-I for non-randomized interventions rely on consistent reporting of core parameters, including detailed exposure data, selection criteria, and comparability between groups. In this case, key information such as the exact type, timing, dose, and proportion of mothers receiving antibiotic prophylaxis was missing or inconsistently reported. This lack of basic data precluded a robust, structured assessment of relevant bias domains. Consequently, no formal tool-based risk of bias assessment was feasible. Instead, the methodological quality of the included studies was evaluated narratively within the framework of the SWiM-analysis guidelines. Although this represents an inherent limitation of the present review, it was unavoidable given the current state of evidence in this field.

**Figure 1:**
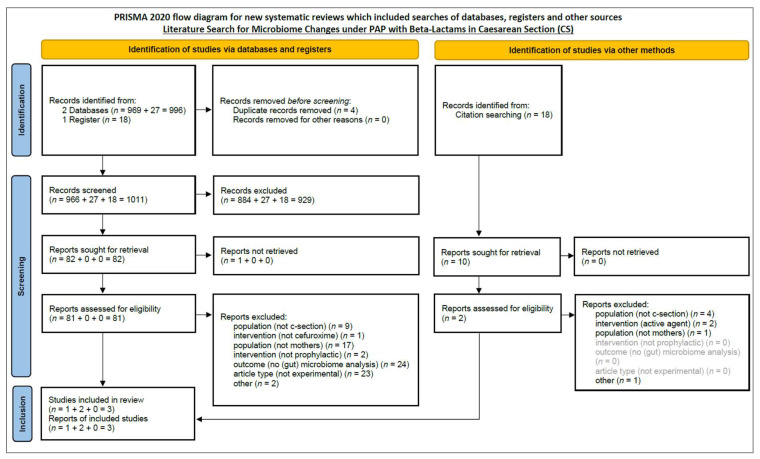
Flow diagram of the selection process according to PRISMA / MECIR standards.

Out of 1011 screened records, three studies met the inclusion criteria and were included in the final analysis (see Figure 1 for details). Details of studies sought for retrieval but excluded after full-text review, along with reasons for exclusion, are provided in Supplementary File S3.

No generative artificial intelligence or automation tools were used for study selection, data analysis, or interpretation. A PRISMA flowchart of the screening and selection process is provided in Figure 1.

## 3. Results

### 3.1. Overview of Included Studies

Three studies met the inclusion criteria and were included in the final analysis. All three reported maternal microbiome data in the context of neonatal-focused research and did not primarily aim to investigate the maternal microbiome. All studies were conducted in Europe (United Kingdom, Sweden, and Spain) and employed molecular biological methods for microbial analysis.

Across studies, sequencing was performed using either shotgun metagenomics via next-generation sequencing (NGS) platforms (Illumina HiSeq2000/2500) or pyrosequencing (Roche/454). While both technologies are suitable for assessing microbial composition, differences in sequencing depth, error rates, and taxonomic resolution restrict direct comparability. Sample sizes varied widely, ranging from 13 to 175 mothers. Caesarean section rates ranged from 15% to 37%, and details about intrapartum antibiotic administration was reported in up to 51% of participants in individual studies.

In one study, stool samples were collected from mothers both before and after delivery, allowing intraindividual analysis of microbial changes across the peripartum period [20]. In the second study, samples were collected only postpartum [21], while in the third, the timing of sampling was not clearly reported [22]. None of the studies explicitly reported maternal microbiome changes in response to antibiotic prophylaxis as a primary outcome. However, relevant data on microbial diversity were available and extracted for this review. Subgroup stratification by delivery mode and specific antibiotic regimens was not possible because essential subgroup data were not reported in sufficient detail, reflecting that mothers were not the primary population of interest in these studies. A detailed summary of study characteristics is provided in Table 1. The graphical abstract summarizes the study rationale, the workflow, and the key findings of this review, including the overall trends for alpha- and beta-diversity shifts.

### 3.2. Alpha-Diversity Metrics

All three included studies reported alpha-diversity parameters, though the selection of metrics and level of detail varied considerably. The most comprehensive data were provided by Vallès et al., who evaluated both taxonomic and functional diversity in maternal stool samples collected before and after delivery [20]. Reported metrics included the Richness Estimator N, Chao1 index, and Shannon index, applied to both taxonomic and predicted functional profiles.

In this review, both taxonomic and functional alpha-diversity metrics are reported. Functional diversity refers to the intra-sample variability in predicted gene functions (e.g., Kyoto Encyclopedia of Genes and Genomes (KEGG) orthologs (KO)), in contrast to taxonomic diversity based on microbial taxa (e.g., operational taxonomic units (OTUs) or genera). Both forms reflect compositional richness and evenness within individual samples but operate at different biological levels.

To assess intraindividual changes, we reanalysed the reported values from Vallès et al. [20] using the Wilcoxon signed-rank test. No statistically significant differences were observed in taxonomic diversity between pre- and post-delivery samples (Richness Estimator N: *p* = 0.216; Chao1 index: *p* = 0.414; Shannon index: *p* = 0.588). However, functional diversity metrics showed significant reductions postpartum (Richness Estimator N: *p* = 0.017; Chao1 index: *p* = 0.002; Shannon index: *p* = 0.017). These findings suggest that microbial functional potential may be more sensitive to peripartum influences, such as surgical stress or antibiotic exposure, than taxonomic composition alone.

Shao et al. [21] reported a single Shannon index value for all mothers combined, without stratification by delivery mode or antibiotic exposure. This reduces interpretability but supports the general range observed by Vallès et al. [20]. Bäckhed et al. [22] reported the number of OTUs only for the vaginal delivery subgroup, without comparable data for Caesarean section or antibiotic-treated groups.

Due to inconsistencies in reporting, lack of stratification, and methodological inconsistencies, direct comparison across studies was hindered. Nonetheless, the available data highlight that functional alpha-diversity metrics may detect biologically relevant microbiome alterations that are not reflected in taxonomic diversity measures. A full compilation of alpha-diversity metrics is presented in Table 2. A complete overview of all extracted metrics and raw data is available in Supplementary File S2.

### 3.3. Beta-Diversity Metrics

Beta-diversity, which assesses differences in microbiome composition between samples or groups, was reported in all three studies using various statistical and distance-based approaches, including Bray–Curtis dissimilarity, ANOSIM, PERMANOVA, and UniFrac analyses. All reported metrics are summarized in Table 3.

Vallès et al. [20] analysed both taxonomic and functional beta-diversity using Gower distances and visualized the results via principal coordinate analysis (PCoA). No significant clustering of pre- versus post-delivery samples was observed (taxonomic: *p* = 0.4500; functional: *p* = 0.4793). ANOSIM results indicated a trend toward reduced beta-diversity postpartum, with lower median Bray–Curtis dissimilarity values after birth, though without statistical significance (taxonomic: *p* = 0.2241; functional: *p* = 0.0798).

Shao et al. [21] analysed maternal stool samples using PERMANOVA on Bray–Curtis dissimilarity matrices. No significant differences in microbial community composition were detected with respect to intrapartum antibiotic prophylaxis (IAP: R^2^ = 0.01492, *p* = 0.8342) or vaginal delivery (VD: R^2^ = 0.04693, *p* = 0.9371). Insufficient metadata prevented analysis of CS as a separate category.

Bäckhed et al. [22] reported genus-level Bray–Curtis dissimilarity indices, showing numerically higher dissimilarity in CS deliveries compared to VD (median [IQR]: 0.495 [0.560–0.430] vs. 0.425 [0.500–0.351]). However, statistical testing of these differences using the Mann–Whitney U test yielded no significant result (*p* = 0.667). Similarly, re-analysis of Bray-Curtis distances based on MetaOTUs yielded a *p*-value of 1.0. Additional data from the same study included unweighted UniFrac distances at KEGG ortholog (KO) level for mother–infant pairs (median: 0.1725 [0.2125–0.1400]). However, no subgroup-specific data for mothers were available, precluding further interpretation.

While beta-diversity was assessed in all studies [20,21,22], methodological divergence and limited subgroup resolution constrain comparability. IAP and delivery mode explained only a minimal proportion of beta-diversity variation (R^2^ < 0.05), indicating negligible group-level effects on maternal microbial composition. The small effect sizes and inconsistent clustering patterns demand closer examination of the antibiotic regimens applied during delivery as potential sources of variability.

### 3.4. Reported Antibiotic Regimen

Details regarding the antibiotic regimens administered during childbirth varied considerably across studies and contributed substantially to between-study heterogeneity (see Table 4).

According to WHO recommendations, all women undergoing Caesarean section should receive PAP to reduce the risk of surgical site infections. Two of the included studies [20,21] reported full compliance with this guideline, administering PAP to 100% of mothers delivering by CS. In contrast, Bäckhed et al. [22] reported only partial adherence, with 10 out of 15 CS patients receiving intrapartum antibiotics. This variability in implementation complicates the interpretation of microbiome outcomes across studies, as inconsistent antibiotic exposure may obscure true associations.

Antibiotic use during VD was less frequent but not negligible, with reported administration rates ranging from 14% to 23%. Importantly, the studies applied different antibiotic agents and regimens. One study employed multiple antibiotic protocols across participants [22], while another did not report the specific agent or dosage used [20]. In the third study [21], no detailed information on antibiotic substances or administration protocols was provided.

This inconsistency in reporting and implementation of antibiotic interventions adds an additional layer of complexity and reduces the comparability of microbiome outcomes. In cases where no detailed documentation of the substance or dose is available, this results in an unquantified antimicrobial exposure at birth—affecting both maternal and neonatal microbial ecosystems. The inability to stratify findings by specific antibiotic type or dosage further restricts causal interpretation regarding the impact of individual agents on maternal gut microbial composition at the time of delivery. This also hampers the systematic comparison of microbial effects in mothers and newborns across studies.

A significant limitation of this survey is the heterogeneity of reported metrics and methods (see Table 2 and Table 3). Two studies [20,21] did not report key microbiome diversity indices as OTUs, and several analyses were not comparable due to differences in sample processing, sequencing methods, and statistical approaches. Except for taxonomic Shannon index, which was reported in two of the three studies [20,21], none of the alpha- or beta-diversity parameters were reported in more than one study. This made direct comparisons between studies impossible. The sparse reporting of alpha- and beta-diversity metrics for both delivery modes, as well as the inconsistent reporting of antibiotic interventions, highlights a substantial gap in the current literature. Notably, only one study assessed differences in beta-diversity before and after birth. Any further comparative analysis is therefore not possible due to a lack of data. Importantly, this lack of standardization restricts our understanding of how antibiotic exposure at birth affects maternal microbial resilience, and how these changes may in turn influence neonatal colonization. Without harmonized metrics and reporting frameworks, the role of the maternal microbiome as a determinant of early-life health remains difficult to quantify.

## 4. Discussion

Despite widespread use of antibiotic prophylaxis in Caesarean sections, its effects on the maternal gut microbiome remain underexplored. Although antibiotic impacts on neonatal microbial development have been extensively studied [23,24,25,26], maternal outcomes have received little attention—despite their potential relevance for recovery, immune function, and maternal–infant microbial continuity [27].

Across the three included studies [20,21,22], maternal microbiome data were reported only secondarily and with substantial variability in methodology, sequencing technology, and reporting standards. The only study that provided intraindividual pre- and post-delivery sampling [20] found significant changes in functional alpha-diversity (Chao1: *p* = 0.002; Shannon: *p* = 0.017), while corresponding taxonomic indices remained non-significant. This suggests that certain aspects of microbial richness may be more sensitive to peripartum perturbations than overall taxonomic structure.

Several mechanisms may explain this discrepancy. First, functional diversity metrics such as Chao1 are more sensitive to subtle changes, particularly among rare taxa, which may be disproportionately affected by antibiotic exposure. Second, taxonomic and functional dimensions of the microbiome follow nonlinear relationships. Keystone species with low relative abundance may carry disproportionate metabolic functions, meaning their loss may not shift taxonomic profiles but still disrupt community function [28]. Third, commonly used taxonomic metrics, such as the Shannon index, may lack the resolution to detect minor compositional changes, while functional measures—capturing gene content or predicted metabolic pathways—may be more responsive. Lastly, selective pressures at the interface of delivery, surgery, and antimicrobial exposure may favour functionally adaptive traits while preserving overall taxonomic structure.

Beta-diversity analyses yielded similarly inconclusive results. All three studies [20,21,22] applied different statistical methods (ANOSIM, PERMANOVA, Bray–Curtis, UniFrac), but none found statistically significant clustering by delivery mode or antibiotic exposure. Notably, R^2^ values in PERMANOVA were below 0.05, indicating that neither delivery mode nor intrapartum antibiotic exposure explained a substantial proportion of interindividual microbial variation. Limited subgroup analysis and absence of pre-intervention sampling further constrain interpretation.

The inconsistent reporting of antibiotic regimens increases these restrictions. While PAP is universally recommended for CS, compliance and documentation varied across studies. One study [22] reported that a third of CS patients received no prophylaxis at all; in another, the specific antibiotic and dose were not disclosed. This results in unquantified antimicrobial exposure at birth, affecting both maternal and neonatal microbial ecosystems and precluding stratification by antibiotic agent or regimen. Without standardization, the clinical implications of microbiome shifts remain difficult to assess. Future studies should therefore adhere to standardized implementation and reporting of PAP, including clear documentation of the antibiotic agent, dosage, timing relative to incision, route of administration, and duration. This is essential to enable meaningful comparisons across studies and to clarify the impact of specific regimens on the maternal microbiome. Moreover, standardized documentation of antibiotic administration is not only critical for research comparability but also for clinical transparency and patient rights. Mothers should be fully informed about any antimicrobial intervention they receive during delivery. Yet, such details are often missing or incomplete in routine discharge summaries. Improving documentation practices would therefore benefit both clinical care and the scientific understanding of perinatal antibiotic exposure.

Moreover, the reviewed studies [20,21,22] lacked a maternal-centred research design. All were originally conceived to investigate neonatal outcomes, with maternal data included as ancillary information. As a consequence, detailed subgroup data by delivery mode and antibiotic regimen were often missing, limiting comparability and interpretability of the available diversity metrics. This lack of standardized reporting and missing stratified subgroup data limit comparability and should be explicitly addressed by future studies. The current situation reflects a broader structural imbalance in perinatal research, where the maternal microbiome is often overlooked despite its potential influence on both immediate maternal health and neonatal microbial colonization. Particularly in the context of rising CS rates [29] and the expanding use of antibiotics in obstetric care, a realignment of research priorities is needed.

Taken together, current evidence suggests that maternal gut microbial function may be altered after CS with PAP, even in the absence of detectable taxonomic change.

Although PAP in CS typically consists of a single preoperative dose administered up to 30 min before skin incision, recent data suggest that even such brief exposures can cause measurable and lasting alterations in the gut microbiome. A pilot study in early-stage melanoma patients undergoing clean surgery found that a single dose of perioperative cefazolin significantly reduced gut microbial diversity and depleted key protective species such as *Akkermansia muciniphila* and *Alistipes communis*. These effects persisted for up to 90 days [30]. These findings underscore the microbiome’s sensitivity to brief antibiotic interventions. Although obstetric settings differ, they support the rationale for investigating postpartum microbial changes during physiologically vulnerable periods such as childbirth. Reduced functional diversity in the maternal gut microbiome may have clinically relevant consequences, as it can affect immune modulation, metabolic homeostasis, and tissue repair processes during the postpartum period. A nested case–control study by Ma et al. [31] demonstrated that dysbiosis during pregnancy can increase the risk of gestational diabetes mellitus (GDM) and contribute to adverse outcomes such as preterm birth or preeclampsia, posing potential risks to both mother and child. Similarly, a recent multi-omics study by Huang et al. [12] showed that altered bile acid profiles during pregnancy can modulate low-grade inflammation, potentially promoting immune imbalance and increasing the risk of the aforementioned complications. These findings underline that functional shifts in the maternal microbiome may influence surgical recovery, metabolic adaptation, and overall postpartum health.

However, methodological inconsistency, missing pre-intervention data, and lack of intermediate timepoints prevent definitive conclusions at present. To close this gap, future studies should focus specifically on mothers. They need to be designed as longitudinal investigations with standardized protocols that specify sampling at key timepoints (e.g., pre-pregnancy, each trimester, intrapartum pre- and post-antibiotic, and postpartum) and stratify by delivery mode. Beyond that, these studies should apply shotgun metagenomic sequencing to enable functional microbiome analyses, report amplicon sequencing variants (ASVs) instead of OTUs, and adhere to standardized reporting of a core set of diversity metrics, including standard statistical data, to improve comparability and reproducibility. Understanding these dynamics is essential—not only for maternal recovery and health, but also for shaping the early microbial environment of the newborn.

## 5. Conclusions

Current evidence on the effects of perioperative antibiotic prophylaxis during Caesarean section on the maternal gut microbiome is limited and methodologically heterogeneous. While no consistent taxonomic shifts have been observed, functional diversity metrics suggest that microbial richness and resilience may be altered postpartum. These findings highlight the need for maternal-centred research designs, standardized methodologies, and better reporting of antibiotic regimens. Future research should therefore adopt maternal-focused, longitudinal study designs with sampling at key timepoints, stratification by delivery mode, functional sequencing methods such as shotgun metagenomics, and standardized reporting of core microbiome metrics to ensure comparability and reproducibility.

Understanding the impact of intrapartum antibiotic exposure on maternal microbial dynamics is essential—not only for maternal recovery, but also for characterizing the early microbial environment to which the newborn is exposed.

## Figures and Tables

**Table 1:** Structural key characteristics of included studies. Study references correspond to [20,21,22].

Publication					Methods		
First Author	Year	Country	Scope	Main Result	Total Study Population	Time of Stool Sampling	Sequencing Method (Platform)
Vallès [20]	2014	Spain	taxonomic and functional gut microbiota succession in infants	two-phase succession driven by solid food; progresses toward maternal microbiota but incomplete by 1 year	26	1 week prior to delivery; 1 year after delivery	Metagenomic Analysis via Pyro-sequencing (Roche/454 sequencing)
Bäckhed [22]	2015	Sweden	dynamics of infant gut microbiome influenced by delivery mode and feeding	cessation of breastfeeding drives functional maturation to an adult-like microbiome	196	2 days after delivery	Shotgun Metagenomic Sequencing via NGS (Illumina Hiseq2000)
Shao [21]	2019	UK	impact of CS on neonatal gut microbiota development	CS disrupts maternal microbial transfer, increases colonization by hospital-associated pathogens	771	before OR after OR during delivery	Shotgun Metagenomic Sequencing via NGS (Illumina Hiseq2500 v4)

Notes: CS: Caesarean section; WGS: Whole Genome Sequencing; NGS: Next Generation Sequencing. Total study population = mothers plus infants.

**Table 2:** Reported alpha-diversity metrics for taxonomic and functional diversity from the included studies. For Vallès et al. (2014), intraindividual pre- vs. post-birth comparisons were available; other studies reported unstratified summary values only. OTU counts reported by Bäckhed et al. (2015) refer to VD patients only. VD = vaginal delivery. Study references correspond to [20,21,22] in the main text.

Publication	Metric	Median (±SE)	IQR	Range (Min–Max)	Comparison	Statistical Test	*p*- Value
Vallès (2014) [20]	Chao1 index (taxonomic)	337.18 (±13.43)	79.86	193.48–512.90	pre- vs. post-birth	Wilcoxon signed-rank	0.414
Vallès (2014) [20]	Shannon index (taxonomic)	3.28 (±0.12)	0.83	1.36–4.66	pre- vs. post-birth	Wilcoxon signed-rank	0.588
Vallès (2014) [20]	Richness Estimator N (taxonomic)	238.82 (±9.44)	55.82	136.38–359.66	pre- vs. post-birth	Wilcoxon signed-rank	0.216
Vallès (2014) [20]	Chao1 index (functional)	95.71 (±0.77)	3.2	89.22–102.1	pre- vs. post-birth	Wilcoxon signed-rank	0.002
Vallès (2014) [20]	Shannon index (functional)	5.77 (±0.01)	0.07	5.63–5.90	pre- vs. post-birth	Wilcoxon signed-rank	0.017
Vallès (2014) [20]	Richness Estimator N (functional)	90.98 (±0.43)	2.24	86.69–95.65	pre- vs. post-birth	Wilcoxon signed-rank	0.017
Bäckhed (2015) [22]	OTUs (taxonomic)	690 (n.r.)	n.r.	n.r.	n.r.	n.a.	n.a.
Shao (2019) [21]	Shannon index (taxonomic)	3.125 (n.r.)	0.4375	2.875–3.3125	n.r.	n.a.	n.a.

Notes: n.r. = not reported by study authors; n.a. = not applicable.

**Table 3:** Reported beta-diversity analyses from the included studies. For Vallès et al. (2014), intraindividual pre- vs. post-birth comparisons indicated a trend toward postpartum reduction, though not significant. Shao et al. (2019) analysed intrapartum antibiotic prophylaxis (IAP) as a separate factor. Bäckhed et al. (2015) reported genus-level dissimilarity for CS vs. VD and unweighted UniFrac distances for mother–infant pairs without subgroup-specific maternal results. CS = Caesarean section; VD = vaginal delivery; IAP = intrapartum antibiotic prophylaxis; KO = KEGG ortholog. Study references correspond to references [20,21,22] in the main text.

Publication	Metric	Comparison Groups	Statistical Test	*p*-Value	Effect Size
Vallès (2014) [20]	ANOSIM (Bray–Curtis, taxonomic)	pre vs. post birth	ANOSIM	0.2241	n.a.
Vallès (2014) [20]	ANOSIM (Bray–Curtis, functional)	pre vs. post birth	ANOSIM	0.0798	n.a.
Vallès (2014) [20]	PCoA (Gower distances, taxonomic)	pre vs. post birth	PCoA	0.45	d > 0.8
Vallès (2014) [20]	PCoA (Gower distances, functional)	pre vs. post birth	PCoA	0.4793	d > 0.8
Bäckhed (2015) [22]	Bray–Curtis (genus level)	CS vs. VD	Mann–Whitney U	0.667	n.a.
Bäckhed (2015) [22]	Bray–Curtis (MetaOTUs)	CS vs. VD	Mann–Whitney U	1	n.a.
Bäckhed (2015) [22]	UniFrac (unweighted, KO level)	mothers vs. infants	n.a.	n.a.	n.a.
Shao (2019) [21]	PERMANOVA (Bray–Curtis)	VD	PERMANOVA	0.9371	R^2^ = 0.04693
Shao (2019) [21]	PERMANOVA (Bray–Curtis)	IAP	PERMANOVA	0.8342	R^2^ = 0.01492

Notes: R^2^ = proportion of variance explained; d = dissimilarity index; n.a. = not applicable.

**Table 4:** Reported intrapartum antibiotic prophylaxis (IAP) regimens in the included studies. Percentages refer to the total number of mothers included per study (total mothers (n)). All regimens were administered intravenously (IV). CS = Caesarean section; VD = vaginal delivery; IAP = intrapartum antibiotic prophylaxis.

Publication	Mothers			Intrapartum Antibiotic Prophylaxis (IAP)					Antibiotic Regimen	
	total mothers (n)	CS (n)	VD (n)	IAP given			IAP not reported (n, %)	control /no IAP (n, %)	IAP during CS delivery (agent and dosing, n)	IAP before any delivery (agent and dosing, n)
				total (n, %)	in CS (n, %)	in VD (n, %)				
Shao 2019 [21]	175	65	110	90	65	25	77	8	not reported	not reported
				(51%)	(37%)	(14%)	(44%)	(5%)		
Bäckhed 2015 [22]	98	15	83	20	10	10	not reported	not reported	1 × 4 g Piperacillin/Tazobactam (*n* = 8) 1 × 600 mg Clindamycin (*n* = 2)	1–4 × 3 g Benzylpenicillin (*n* = 12) 1 × 900 mg Clindamycin (*n* = 1)
				(20%)	(10%)	(10%)	(not applicable)	(not applicable)		
Vallès 2014 [20]	13	3	10	6	3	3	0	7	Amoxicillin, dosage not reported (*n* = 3)	Benzylpenicillin, dosage not reported (*n* = 1); Amoxicillin, dosage not reported (*n* = 2)
				(46%)	(23%)	(23%)	(0%)	(54%)		

Note: In this table, ‘IAP’ refers to all intrapartum antibiotic prophylaxis, including perioperative antibiotic prophylaxis (PAP) given during CS and any intrapartum antibiotic use during VD. Percentages for ‘IAP Given’, ‘Control/No IAP’ and ‘Not Reported’ sum to 100% per study.

## Data Availability

All data extracted and analysed in this review are derived from published sources and are presented in the manuscript or Supplementary Materials. Additional datasets generated during the review process, including the full literature search output, are available from the corresponding author upon reasonable request.

## Supplementary Materials

The following supporting information can be downloaded at: https://www.mdpi.com/article/10.3390/jcm14145104/s1, Supplementary File S1: Search_strategy; Supplementary File S2: Extracted_data; Supplementary File S3: Excluded_studies; Supplementary File S4: PRISMA_2020_Checklist; Supplementary File S5: PRISMA-S_Checklist.

## Abbreviations

The following abbreviations are used in this manuscript:

ANOSIM	Analysis of Similarities
ASV	Amplicon Sequencing Variant
CS	Caesarean section
IAP	intrapartum antibiotic prophylaxis
ICTRP	International Clinical Trials Registry Platform
IV	intravenously
IQR	Interquartile Range
KEGG	Kyoto Encyclopedia of Genes and Genomes
KO	KEGG Ortholog
MECIR	Methodological Expectations of Cochrane Intervention Reviews
NGS	Next-Generation Sequencing
OTU	Operational Taxonomic Unit
PAP	Perioperative Antibiotic Prophylaxis
PERMANOVA	Permutational Multivariate Analysis of Variance
PICO	Population, Intervention, Comparison, Outcome
PCoA	Principal Coordinates Analysis
PRISMA	Preferred Reporting Items for Systematic Reviews and Meta-Analyses
RoB	Risk of Bias
SE	Standard Error
SSI	Surgical Site Infection
SWiM	Synthesis Without Meta-Analysis
VD	Vaginal Delivery
WHO	World Health Organization
