# Supplementary material for: Impact of Perioperative Antibiotic Prophylaxis in Caesarean Section on the Maternal Gut Microbiome: A Systematic Review
Source: J Clin Med. 2025 Jul 18;14(14):5104. doi: 10.3390/jcm14145104 (PMC12295807; doi:10.3390/jcm14145104)
Supplement: Supplementary file 1 [file jcm-14-05104-s001.zip › Supplementary File_S1_search_strategy.pdf]

Supplement A.1 – Full search strategy: Maternal Microbiome (Feles 2025)

MedLine via PubMed (Cefuroxime)

Search number	Query	Sort By	Filters	Search Details	Results	Time
1	"Pregnancy"[Mesh]	Most Recent		"Pregnancy"[MeSH Terms]	992,465	05:40:08
2	pregnant[Title/Abstract]			"pregnant"[Title/Abstract]	213,226	05:40:22
3	women[Title/Abstract]			"women"[Title/Abstract]	1,157,866	05:40:34
4	birth[Title/Abstract]			"birth"[Title/Abstract]	345,842	05:40:56
5	childbirth[Title/Abstract]			"childbirth"[Title/Abstract]	22,53	05:41:28
6	delivery[Title/Abstract]			"delivery"[Title/Abstract]	563,399	05:41:41
7	"Cesarean Section"[Mesh]	Most Recent		"Cesarean Section"[MeSH Terms]	52,145	05:42:12
8	"Delivery, Obstetric"[Mesh]	Most Recent		"delivery, obstetric"[MeSH Terms]	90,036	05:42:45
9	c-section[Title/Abstract]			"c-section"[Title/Abstract]	1,726	05:43:05
10	c section[Title/Abstract]			"c section"[Title/Abstract]	1,726	05:43:25
11	caesarean section[Title/Abstract]			"caesarean section"[Title/Abstract]	19,376	05:43:41
12	cesarean section[Title/Abstract]			"cesarean section"[Title/Abstract]	30,981	05:43:58
13	#1 OR #2 OR #3 OR #4 OR #5 OR #6 OR #7 OR #8 OR #9 #10 OR #11 OR #12			(("Pregnancy"[MeSH Terms] OR "pregnant"[Title/Abstract] OR "women"[Title/Abstract] OR "birth"[Title/Abstract] OR "childbirth"[Title/Abstract] OR "delivery"[Title/Abstract] OR "Cesarean Section"[MeSH Terms] OR "delivery, obstetric"[MeSH Terms] OR "c-section"[Title/Abstract]) AND "c-section"[Title/Abstract]) OR "caesarean section"[Title/Abstract] OR "Cesarean Section"[Title/Abstract]	50,188	05:44:34
14	"Cefuroxime"[Mesh]	Most Recent		"Cefuroxime"[MeSH Terms]	2,308	05:45:04
15	cephuroxime[Title/Abstract]			"cephuroxime"[Title/Abstract]	14	05:45:22
16	Zinacef[Title/Abstract]			"Zinacef"[Title/Abstract]	30	05:45:36
17	Ketocef[Title/Abstract]			"Ketocef"[Title/Abstract]	5	05:45:52
18	(("Antibiosis"[Mesh]) OR "Antibiotic Prophylaxis"[Mesh]) OR "prevention and control" [Subheading]			"Antibiosis"[MeSH Terms] OR "Antibiotic Prophylaxis"[MeSH Terms] OR "prevention and control"[MeSH Subheading]	1,447,082	05:46:40
19	prevention[Title/Abstract]			"prevention"[Title/Abstract]	686,945	05:47:03
20	prophylactic[Title/Abstract]			"prophylactic"[Title/Abstract]	89,739	05:47:22
21	chemoprophylaxis[Title/Abstract]			"chemoprophylaxis"[Title/Abstract]	6,654	05:47:36

Supplement A.1 – Full search strategy: Maternal Microbiome (Feles 2025)

22	chemoprevention[Title/Abstract]			"chemoprevention"[Title/Abstract]	12,389	05:47:50
23	#14 OR #15 OR #16 OR #17 OR #18 OR #19 OR #20 OR #21 OR #22			"Cefuroxime"[MeSH Terms] OR "cephuroxime"[Title/Abstract] OR "Zinacef"[Title/Abstract] OR "Ketocef"[Title/Abstract] OR "Antibiosis"[MeSH Terms] OR "Antibiotic Prophylaxis"[MeSH Terms] OR "prevention and control"[MeSH Subheading] OR "prevention"[Title/Abstract] OR "prophylactic"[Title/Abstract] OR "chemoprophylaxis"[Title/Abstract] OR "chemoprevention"[Title/Abstract]	1,918,425	05:48:09
24	"Microbiota"[Mesh] OR "Gastrointestinal Microbiome"[Mesh]			"Microbiota"[MeSH Terms] OR "Gastrointestinal Microbiome"[MeSH Terms]	70,371	05:48:28
25	microbiome[Title/Abstract]			"microbiome"[Title/Abstract]	50,491	05:48:47
26	stool sample[Title/Abstract]			"stool sample"[Title/Abstract]	1,665	05:49:01
27	fecal sample[Title/Abstract]			"fecal sample"[Title/Abstract]	999	05:49:17
28	feces[Title/Abstract]			"feces"[Title/Abstract]	35,506	05:49:29
29	#24 OR #25 OR #26 OR #27 OR #28			"Microbiota"[MeSH Terms] OR "Gastrointestinal Microbiome"[MeSH Terms] OR "microbiome"[Title/Abstract] OR "stool sample"[Title/Abstract] OR "fecal sample"[Title/Abstract] OR "feces"[Title/Abstract]	129,194	05:49:44
30	#13 AND #23 AND #29	Most Recent		((("Pregnancy"[MeSH Terms] OR "pregnant"[Title/Abstract] OR "women"[Title/Abstract] OR "birth"[Title/Abstract] OR "childbirth"[Title/Abstract] OR "delivery"[Title/Abstract] OR "Cesarean Section"[MeSH Terms] OR "delivery, obstetric"[MeSH Terms] OR "c-section"[Title/Abstract]) AND "c-section"[Title/Abstract]) OR "caesarean section"[Title/Abstract] OR "Cesarean Section"[Title/Abstract]) AND ("Cefuroxime"[MeSH Terms] OR "cephuroxime"[Title/Abstract] OR "Zinacef"[Title/Abstract] OR "Ketocef"[Title/Abstract] OR ("Antibiosis"[MeSH Terms] OR "Antibiotic Prophylaxis"[MeSH Terms] OR "prevention and control"[MeSH Subheading]) OR "prevention"[Title/Abstract] OR "prophylactic"[Title/Abstract] OR "chemoprophylaxis"[Title/Abstract] OR "chemoprevention"[Title/Abstract]) AND ("Microbiota"[MeSH Terms] OR "Gastrointestinal Microbiome"[MeSH Terms] OR "microbiome"[Title/Abstract] OR "stool sample"[Title/Abstract] OR "fecal sample"[Title/Abstract] OR "feces"[Title/Abstract])	54	05:50:43

Supplement A.1 – Full search strategy: Maternal Microbiome (Feles 2025)

search conducted on 26.11.2025 in MedLine via PubMed

Supplement A.1 – Full search strategy: Maternal Microbiome (Feles 2025)

MedLine via PubMed (Beta-lactams)

Search number	Query	Sort By	Filters	Search Details	Results	Time
1	"Pregnancy"[Mesh]	Most Recent		"Pregnancy"[MeSH Terms]	1,006,017	07:59:25
2	pregnant[Title/Abstract]	Most Recent		"pregnant"[Title/Abstract]	218,741	08:00:11
3	women[Title/Abstract]	Most Recent		"women"[Title/Abstract]	1,185,251	08:00:33
4	birth[Title/Abstract]	Most Recent		"birth"[Title/Abstract]	354,147	08:00:59
5	childbirth[Title/Abstract]	Most Recent		"childbirth"[Title/Abstract]	23,277	08:01:28
6	delivery[Title/Abstract]	Most Recent		"delivery"[Title/Abstract]	581,436	08:01:50
7	"Cesarean Section"[Mesh]	Most Recent		"Cesarean Section"[MeSH Terms]	53,051	08:02:25
8	"Delivery, Obstetric"[Mesh]	Most Recent		"delivery, obstetric"[MeSH Terms]	91,258	08:02:55
9	c-section[Title/Abstract]	Most Recent		"c-section"[Title/Abstract]	1,806	08:13:25
10	c section[Title/Abstract]	Most Recent		"c section"[Title/Abstract]	1,806	08:13:53
11	caesarean section[Title/Abstract]	Most Recent		"caesarean section"[Title/Abstract]	19,728	08:15:03
12	cesarean section[Title/Abstract]	Most Recent		"cesarean section"[Title/Abstract]	31,737	08:15:31
13	#1 OR #2 OR #3 OR #4 OR #5 OR #6 OR #7 OR #8 OR #9 OR #10 OR #11 OR #12	Most Recent		"Pregnancy"[MeSH Terms] OR "pregnant"[Title/Abstract] OR "women"[Title/Abstract] OR "birth"[Title/Abstract] OR "childbirth"[Title/Abstract] OR "delivery"[Title/Abstract] OR "Cesarean Section"[MeSH Terms] OR "delivery, obstetric"[MeSH Terms] OR "c-section"[Title/Abstract] OR "c-section"[Title/Abstract] OR "caesarean section"[Title/Abstract] OR "Cesarean Section"[Title/Abstract]	2,573,621	08:17:26
14	"beta-Lactams"[Mesh]	Most Recent		"beta-Lactams"[MeSH Terms]	139,274	08:51:20
15	beta-lactam*[Title/Abstract]	Most Recent		"beta lactam*"[Title/Abstract]	52,752	08:59:12
16	betalactam*[Title/Abstract]	Most Recent		"betalactam*"[Title/Abstract]	48,213	08:59:36
17	"Antibiosis"[Mesh]	Most Recent		"Antibiosis"[MeSH Terms]	5,058	09:04:17
18	"Antibiotic Prophylaxis"[Mesh]	Most Recent		"Antibiotic Prophylaxis"[MeSH Terms]	15,501	09:05:35
19	prophylaxis[Title/Abstract]	Most Recent		"prophylaxis"[Title/Abstract]	113,783	09:08:24
20	prophylactic[Title/Abstract]	Most Recent		"prophylactic"[Title/Abstract]	91,575	09:08:43
21	chemoprophylaxis[Title/Abstract]	Most Recent		"chemoprophylaxis"[Title/Abstract]	6,734	09:09:32
22	chemoprevention[Title/Abstract]	Most Recent		"chemoprevention"[Title/Abstract]	12,581	09:09:55

Supplement A.1 – Full search strategy: Maternal Microbiome (Feles 2025)

23	#14 OR #15 OR #16 OR #17 OR #18 OR #19 OR #20 OR #21 OR #22	Most Recent		"beta-Lactams"[MeSH Terms] OR "beta lactam*"[Title/Abstract] OR "betalactam*"[Title/Abstract] OR "Antibiosis"[MeSH Terms] OR "Antibiotic Prophylaxis"[MeSH Terms] OR "prophylaxis"[Title/Abstract] OR "prophylactic"[Title/Abstract] OR "chemoprophylaxis"[Title/Abstract] OR "chemoprevention"[Title/Abstract]	380,055	09:17:55
24	"Microbiota"[Mesh]	Most Recent		"Microbiota"[MeSH Terms]	74,935	09:18:29
25	microbiome[Title/Abstract]	Most Recent		"microbiome"[Title/Abstract]	55,308	09:18:50
26	"Feces"[Mesh]	Most Recent		"Feces"[MeSH Terms]	108,192	09:19:48
27	stool sample[Title/Abstract]	Most Recent		"stool sample"[Title/Abstract]	1,716	09:20:10
28	fecal sample[Title/Abstract]	Most Recent		"fecal sample"[Title/Abstract]	1,036	09:20:35
29	"Gastrointestinal Tract"[Mesh]	Most Recent		"Gastrointestinal Tract"[MeSH Terms]	692,999	09:22:05
30	#24 OR #25 OR #26 OR #27 OR #28 OR #29	Most Recent		"Microbiota"[MeSH Terms] OR "microbiome"[Title/Abstract] OR "Feces"[MeSH Terms] OR "stool sample"[Title/Abstract] OR "fecal sample"[Title/Abstract] OR "Gastrointestinal Tract"[MeSH Terms]	864,42	09:23:16
31	#13 AND #23 AND #30	Most Recent		("Pregnancy"[MeSH Terms] OR "pregnant"[Title/Abstract] OR "women"[Title/Abstract] OR "birth"[Title/Abstract] OR "childbirth"[Title/Abstract] OR "delivery"[Title/Abstract] OR "Cesarean Section"[MeSH Terms] OR "delivery, obstetric"[MeSH Terms] OR "c-section"[Title/Abstract] OR "c-section"[Title/Abstract] OR "caesarean section"[Title/Abstract] OR "Cesarean Section"[Title/Abstract]) AND ("beta-Lactams"[MeSH Terms] OR "beta lactam*"[Title/Abstract] OR "betalactam*"[Title/Abstract] OR "Antibiosis"[MeSH Terms] OR "Antibiotic Prophylaxis"[MeSH Terms] OR "prophylaxis"[Title/Abstract] OR "prophylactic"[Title/Abstract] OR "chemoprophylaxis"[Title/Abstract] OR "chemoprevention"[Title/Abstract]) AND ("Microbiota"[MeSH Terms] OR "microbiome"[Title/Abstract] OR "Feces"[MeSH Terms] OR "stool sample"[Title/Abstract] OR "fecal sample"[Title/Abstract] OR "Gastrointestinal Tract"[MeSH Terms])	915	09:23:46
search conducted on 26.11.2024 in MedLine via PubMed						

Supplement A.1 – Full search strategy: Maternal Microbiome (Feles 2025)

Cochrane Database (Cefuroxime)

Search Name:		
Date Run:	26.11.2024	
Comment:		
ID	Search	Hits
#1	MeSH descriptor: [Pregnancy] explode all trees	24374
#2	MeSH descriptor: [Pregnant Women] explode all trees	458
#3	MeSH descriptor: [Parturition] explode all trees	526
#4	MeSH descriptor: [Parturition] explode all trees	526
#5	MeSH descriptor: [Delivery, Obstetric] explode all trees	5659
#6	MeSH descriptor: [Cesarean Section] explode all trees	3416
#7	MeSH descriptor: [Cesarean Section] explode all trees	3416
#8	#1 OR #2 OR #3 OR #4 OR #5 OR #6 OR #7	24746
#9	MeSH descriptor: [Cefuroxime] explode all trees	468
#10	MeSH descriptor: [Antibiosis] explode all trees	30
#11	MeSH descriptor: [Chemoprevention] explode all trees	1801
#12	MeSH descriptor: [Antibiotic Prophylaxis] explode all trees	1339
#13	peri-operative	2441
#14	perioperative	22811
#15	#9 OR #10 OR #11 OR #12 OR #13 OR #14	24920
#16	MeSH descriptor: [Microbiota] explode all trees	1190
#17	MeSH descriptor: [Gastrointestinal Microbiome] explode all trees	915
#18	MeSH descriptor: [Feces] explode all trees	3164
#19	stool	9796
#20	stool sample	1241
#21	fecal sample	1173
#22	faecal sample	1172

#23	#16 OR #17 OR #18 OR #19 OR #20 OR #21 OR #22	13291
#24	#8 AND #15 AND #23	9

Supplement A.1 – Full search strategy: Maternal Microbiome (Feles 2025)

Cochrane Database (Beta-lactams)

Search Name:		
Date Run:	26.11.2024	
Comment:		
ID	Search	Hits
#1	MeSH descriptor: [Pregnancy] explode all trees	31435
#2	MeSH descriptor: [Pregnant Women] explode all trees	774
#3	MeSH descriptor: [Parturition] explode all trees	741
#4	MeSH descriptor: [Delivery, Obstetric] explode all trees	7260
#5	MeSH descriptor: [Cesarean Section] explode all trees	4662
#6	#1 OR #2 OR #3 OR #4 OR #5	32356
#7	MeSH descriptor: [Antibiosis] explode all trees	34
#8	MeSH descriptor: [Chemoprevention] explode all trees	2424
#9	MeSH descriptor: [Antibiotic Prophylaxis] explode all trees	1778
#10	MeSH descriptor: [beta-Lactams] explode all trees	10556
#11	peri-operative	2735
#12	perioperative	26679
#13	#7 OR #8 OR #9 OR #10 OR #11 OR #12	38676
#14	MeSH descriptor: [Microbiota] explode all trees	1797
#15	MeSH descriptor: [Gastrointestinal Microbiome] explode all trees	1346
#16	MeSH descriptor: [Feces] explode all trees	3864
#17	stool	11365
#18	stool sample	1464
#19	fecal sample	1386
#20	faecal sample	1384
#21	#14 OR #15 OR #16 OR #17 OR #18 OR #19 OR #20	15860
#22	#6 AND #13 AND #21	18

Supplement A.1 – Full search strategy: Maternal Microbiome (Feles 2025)

ICTRP (Caesarean section)

Search Name:		
Date Run:	26.11.2024	
Comment:		
ID	Search	Hits
#1	"Caesarean Section" OR "C-Section" OR "C Section" OR "Cesarean section"	
#2	microbiome	
#3	#1 AND #2	18
synonyms		
ceasarean section	abdominal deliveries; abdominal delivery; caesarean section; cesarean sections; C section (OB); C sections (OB); postcesarean section	
c section	c section	
c%section	c%section	
cesarean section	abdominal deliveries; abdominal delivery; caesarean section; cesarean sections; C section (OB); C sections (OB);	
microbiome	microbial communities; microbial community; microbial community composition; microbial community compositions; microbial community structure; microbial community structures; microbiome; microbiotas	
