# Supplementary material for: Impact of Perioperative Antibiotic Prophylaxis in Caesarean Section on the Maternal Gut Microbiome: A Systematic Review
Source: J Clin Med. 2025 Jul 18;14(14):5104. doi: 10.3390/jcm14145104 (PMC12295807; doi:10.3390/jcm14145104)
Supplement: Supplementary file 1 [file jcm-14-05104-s001.zip › Supplementary File_S3_excluded_studies.pdf]

Supplement A.3 – Excluded Studies: Maternal Microbiome (Feles 2025)

#	Authors	Year	Title	Journal	PMID	Reason for exclusion
1	Shankar A, Das DJ, Nayar S, Thomas S.	2023	Deciphering the effect of maternal postpartum antibiotic prophylaxis on the infant gut microbiome: a whole metagenomic analysis	Future Microbiol.	37204286	population (not mothers)
2	Manara S, Selma-Royo M, Huang KD, Asnicar F, Armanini F, Blanco-Miguez A, Cumbo F, Golzato D, Manghi P, Pinto F, Valles-Colomer M, Amoroso L, Corrias MV, Ponzoni M, Raffaetà R, Cabrera-Rubio R, Olcina M, Pasolli E, Collado MC, Segata N.	2023	Maternal and food microbial sources shape the infant microbiome of a rural Ethiopian population	Curr Biol.	37116481	intervention (not prophylactic)
3	Moore J, Navarro S, McCarthy K, Rashid RB, Phipps S, Amoako A, Callaway L, Eley V.	2023	State of the art: Intrapartum antibiotics in cesarean section, the infant microbiota and allergic diseases	Acta Obstet Gynecol Scand.	37067195	article type (not experimental)
4	Kuhr K, Axelsson PB, Andersen BR, Ammitzbøll ILA, Clausen TD, Løkkegaard ECL.	2022	Postoperative infections after non-elective cesarean section - a retrospective cohort study of prevalence and risk factors at a single center in Denmark administering prophylactic antibiotics after cord clamping	BMC Pregnancy Childbirth.	36528589	outcome (no (gut) microbiome analysis)
5	Propst K, Yao M, Hickman LC.	2023	Impact of peripartum antibiotics on wound complications in women with obstetric anal sphincter injury	Int J Gynaecol Obstet.	36306399	outcome (no (gut) microbiome analysis)
6	Jokela R, Korpela K, Jian C, Dikareva E, Nikkonen A, Saisto T, Skogberg K, de Vos WM, Kolho KL, Salonen A.	2022	Quantitative insights into effects of intrapartum antibiotics and birth mode on infant gut microbiota in relation to well-being during the first year of life	Gut Microbes.	36174236	population (not mothers)
7	Hanson L, VandeVusse L, Forgie M, Malloy E, Singh M, Scherer M, Kleber D, Dixon J, Hryckowian AJ, Safdar N.	2023	A randomized controlled trial of an oral probiotic to reduce antepartum group B Streptococcus colonization and gastrointestinal symptoms	Am J Obstet Gynecol MFM.	36108911	outcome (no (gut) microbiome analysis)

#	Authors	Year	Title	Journal	PMID	Reason for exclusion
8	Obuchowska A, Gorczyca K, Standyło A, Obuchowska K, Kimber-Trojnar Ż, Wierzchowska-Opoka M, Leszczyńska-Gorzelak B.	2022	Effects of Probiotic Supplementation during Pregnancy on the Future Maternal Risk of Metabolic Syndrome	Int J Mol Sci.	35897822	article type (not experimental)
9	Black M, Kothari A, Chawla G, Pelecanos A, Zahumensky A, McDermott L, O'Connor H, Kalma B, Eley V.	2023	Attitudes and awareness of Australian women regarding peripartum antibiotic use: A multicentre survey	Aust N Z J Obstet Gynaecol.	35856134	outcome (no (gut) microbiome analysis)
10	Ragan MV, Wala SJ, Goodman SD, Bailey MT, Besner GE.	2022	Next-Generation Probiotic Therapy to Protect the Intestines From Injury	Front Cell Infect Microbiol.	35837474	population (not mothers)
11	de Lauzanne A, Sreng N, Foucaud E, Sok T, Chon T, Yem C, Hak V, Heng S, Soda M, Gouali M, Nadimpalli M, Inghammar M, Rabenandrasana MAN, Collard JM, Vray M, Hello SL, Kerleguer A, Piola P, Delarocque-Astagneau E, Guillemot D, Huynh BT, Borand L	2022	Prevalence and factors associated with faecal carriage of extended-spectrum β-lactamase-producing Enterobacterales among peripartum women in the community in Cambodia	J Antimicrob Chemother.	35794710	intervention (not beta-lactam antibiotic)
12	Barg M, Rotem R, Weintraub AY, Grisaru-Granovsky S, Michaelson-Cohen R, Rottenstreich M.	2023	Use of antibiotics in women undergoing correction of an obstetric anal sphincter injury: Results from a national Israeli survey	Int J Gynaecol Obstet.	35617218	population (not c-section)
13	Nadeau HCG, Bisson C, Chen X, Zhao YD, Williams M, Edwards RK.	2022	Vaginal-perianal or vaginal-perineal compared with vaginal-rectal culture-based screening for Group B Streptococci (GBS) colonization during the third trimester of pregnancy: a systematic review and meta-analysis	BMC Pregnancy Childbirth.	35287615	article type (not experimental)
14	Nguyen MH, Fornes R, Kamau N, Danielsson H, Callens S, Fransson E, Engstrand L, Bruyndonckx R, Brusselaers N.	2022	Antibiotic use during pregnancy and the risk of preterm birth: a population-based Swedish cohort study	J Antimicrob Chemother.	35233608	outcome (no (gut) microbiome analysis)
15	Bossung V, Lupatsii M, Dashdorj L, Tassiello O, Jonassen S, Pagel J, Demmert M, Wolf EA, Rody A, Waschina S, Graspeuntner S, Rupp J, Härtel C.	2022	Timing of antimicrobial prophylaxis for cesarean section is critical for gut microbiome development in term born infants	Gut Microbes.	35184691	population (not mothers)

Supplement A.3 – Excluded Studies: Maternal Microbiome (Feles 2025)

#	Authors	Year	Title	Journal	PMID	Reason for exclusion
16	Reyman M, van Houten MA, Watson RL, Chu MLJN, Arp K, de Waal WJ, Schiering I, Plötz FB, Willems RJL, van Schaik W, Sanders EAM, Bogaert D.	2022	Effects of early-life antibiotics on the developing infant gut microbiome and resistome: a randomized trial	Nat Commun.	35173154	population (not mothers)
17	Prescott S, Dreisbach C, Baumgartel K, Koerner R, Gyamfi A, Canellas M, St Fleur A, Henderson WA, Trinchieri G.	2021	Impact of Intrapartum Antibiotic Prophylaxis on Offspring Microbiota	Front Pediatr.	34956974	article type (not experimental)
18	Su Y, Gan XP, Li FF, Zhang DY, Chen L, Cao YN, Qiu HH, Cheng DC, Zu JF, Liu WY, Wang HK, Xu XM.	2021	Effect of exposure to antibiotics on the gut microbiome and biochemical indexes of pregnant women	BMJ Open Diabetes Res Care.	34732397	intervention (not prophylactic)
19	Kanoute A, Gare J, Meda N, Viennot S, Tramini P, Fraticelli L, Carrouel F, Bourgeois D.	2021	Effect of Oral Prophylactic Measures on the Occurrence of Pre-Eclampsia (OP-PE) in High-Risk Pregnant Women: A Cluster Randomized Controlled Trial	Methods Protoc.	34564307	population (not c-section)
20	Oktaviani Sulikah SR, Hasanah M, Setyarini W, Parathon H, Kitagawa K, Nakanishi N, Nomoto R, Osawa K, Kinoshita S, Hirai I, Shirakawa T, Kuntaman K.	2022	Occurrence of Carriage of Multidrug Resistant Enterobacteriaceae among Pregnant Women in the Primary Health Center and Hospital Setting in Surabaya, Indonesia	Microb Drug Resist.	34348048	outcome (no (gut) microbiome analysis)
21	Hui Y, Smith B, Mortensen MS, Krych L, Sørensen SJ, Greisen G, Krogfelt KA, Nielsen DS.	2021	The effect of early probiotic exposure on the preterm infant gut microbiome development	Gut Microbes.	34264803	population (not mothers)
22	Garcia VR.	2021	Impact of Intrapartum Antibiotic Prophylaxis for Group B Streptococcus on the Term Infant Gut Microbiome: A State of the Science Review	J Midwifery Womens Health.	34114318	article type (not experimental)
23	Di Gesù CM, Matz LM, Buffington SA.	2021	Diet-induced dysbiosis of the maternal gut microbiome in early life programming of neurodevelopmental disorders	Neurosci Res.	33992660	article type (not experimental)

Supplement A.3 – Excluded Studies: Maternal Microbiome (Feles 2025)

#	Authors	Year	Title	Journal	PMID	Reason for exclusion
24	Ainonen S, Tejesvi MV, Mahmud MR, Paalanne N, Pokka T, Li W, Nelson KE, Salo J, Renko M, Vänni P, Pirttilä AM, Tapiainen T.	2022	Antibiotics at birth and later antibiotic courses: effects on gut microbiota	Pediatr Res.	33824448	population (not mothers)
25	Imoto N, Kano C, Aoyagi Y, Morita H, Amanuma F, Maruyama H, Nojiri S, Hashiguchi N, Watanabe S.	2021	Administration of β-lactam antibiotics and delivery method correlate with intestinal abundances of Bifidobacteria and Bacteroides in early infancy, in Japan	Sci Rep.	33737648	population (not mothers)
26	Cox CK, Bugosh MD, Fenner DE, Smith R, Swenson CW.	2022	Antibiotic use during repair of obstetrical anal sphincter injury: a quality improvement initiative	Int J Gynaecol Obstet.	33507531	population (not c-section)
27	Attali E, Kern G, Reicher L, Fouks Y, Gamzu R, Yogev Y, Many A.	2021	Early Preterm meconium stained amniotic fluid is an independent risk factor for peripartum maternal bacteremia	Eur J Obstet Gynecol Reprod Biol.	33421814	article type (not experimental)
28	Sommerstein R, Marschall J, Atkinson A, Surbek D, Dominguez-Bello MG, Troillet N, Widmer AF	2020	Antimicrobial prophylaxis administration after umbilical cord clamping in cesarean section and the risk of surgical site infection: a cohort study with 55,901 patients	Antimicrob Resist Infect Control.	33349269	outcome (no (gut) microbiome analysis)
29	Zietek M, Szczuko M, Celewicz Z, Kordek A.	2020	Perinatal factors affecting the gut microbiota - are they preventable?	Ginekol Pol.	33301166	article type (not experimental)
30	Cheng R, Guo J, Zhang Y, Cheng G, Qian W, Wan C, Li M, Marotta F, Shen X, He F.	2021	Impacts of ceftriaxone exposure during pregnancy on maternal gut and placental microbiota and its influence on maternal and offspring immunity in mice	Exp Anim.	33268669	other
31	McCoy JA, Elovitz MA, Alby K, Koelper NC, Nissim I, Levine LD.	2020	Association of Obesity With Maternal and Cord Blood Penicillin Levels in Women With Group B Streptococcus Colonization	Obstet Gynecol.	32925625	outcome (no (gut) microbiome analysis)

#	Authors	Year	Title	Journal	PMID	Reason for exclusion
32	Said M, Dangor Y, Mbelle N, Madhi SA, Kwatra G, Ismail F.	2020	Antimicrobial susceptibility and serotype distribution of Streptococcus agalactiae rectovaginal colonising isolates from pregnant women at a tertiary hospital in Pretoria, South Africa: An observational descriptive study	S Afr Med J.	32880270	population (not c-section)
33	Smith A, Anandan S, Veeraraghavan B, Thomas N.	2020	Colonization of the Preterm Neonatal Gut with Carbapenem-resistant Enterobacteriaceae and Its Association with Neonatal Sepsis and Maternal Gut Flora	J Glob Infect Dis.	32773998	population (not c-section)
34	Steer PJ, Russell AB, Kochhar S, Cox P, Plumb J, Gopal Rao G.	2020	Group B streptococcal disease in the mother and newborn-A review	Eur J Obstet Gynecol Reprod Biol.	32586597	article type (not experimental)
35	Rotem R, Mastrolia SA, Rottenstreich M, Yohay D, Weintraub AY.	2020	The use of metronidazole in women undergoing obstetric anal sphincter injuries: a systematic review of the literature	Arch Gynecol Obstet.	32564129	article type (not experimental)
36	Zanini da Rocha J, Feltraco J, Radin V, Vitola Gonçalves C, Almeida da Silva PE, Von Groll A.	2020	Streptococcus agalactiae colonization and screening approach in high-risk pregnant women in southern Brazil	J Infect Dev Ctries.	32379709	outcome (no (gut) microbiome analysis)
37	Khazaei Z, Ghorbani P, Namaei MH, Rezaei Y, Yousefi M.	2020	Prevalence of Escherichia coli K1 Rectovaginal Colonization Among Pregnant Women in Iran: Virulence Factors and Antibiotic Resistance Properties	Microb Drug Resist.	32354254	population (not c-section)
38	Jisuvei SC, Osoti A, Njeri MA	2020	Prevalence, antimicrobial susceptibility patterns, serotypes and risk factors for group B streptococcus rectovaginal isolates among pregnant women at Kenyatta National Hospital, Kenya	BMC Infect Dis.	32321444	outcome (no (gut) microbiome analysis)
39	McLaren RA, Atallah F, Minkoff H.	2020	Antibiotic Prophylaxis Trials in Obstetrics: A Call for Pediatric Collaboration	AJP Rep.	32309017	article type (not experimental)

Supplement A.3 – Excluded Studies: Maternal Microbiome (Feles 2025)

#	Authors	Year	Title	Journal	PMID	Reason for exclusion
40	Zhou P, Zhou Y, Liu B, Jin Z, Zhuang X, Dai W, Yang Z, Feng X, Zhou Q, Liu Y, Xu X, Zhang L.	2020	Perinatal Antibiotic Exposure Affects the Transmission between Maternal and Neonatal Microbiota and Is Associated with Early-Onset Sepsis	mSphere.	32075882	outcome (no (gut) microbiome analysis)
41	Masuda C, Ferolin SK, Masuda K, Smith C, Matsui M.	2020	Evidence-based intrapartum practice and its associated factors at a tertiary teaching hospital in the Philippines, a descriptive mixed-methods study	BMC Pregnancy Childbirth.	32024504	outcome (no (gut) microbiome analysis)
42	Moron AF.	2020	Author's reply re: Infant microbiota and antibiotic prophylaxis in caesarean delivery	BJOG.	31872569	article type (not experimental)
43	Campo CH, Martínez MF, Otero JC, Rincón G.	2019	Vagino-rectal colonization prevalence by Streptococcus agalactiae and its susceptibility profile in pregnant women attending a third-level hospital	Biomedica.	31860180	outcome (no (gut) microbiome analysis)
44	Winther ACR, Axelsson PB, Clausen TD, Løkkegaard ECL.	2020	Re: Infant microbiota and antibiotic prophylaxis in caesarean delivery	BJOG.	31850610	article type (not experimental)
45	Szylit NA, Malburg FL, Piccinato CA, Ferreira LAP, Podgaec S, Zlotnik E.	2019	Prevalence of rectovaginal colonization by group B Streptococcus in pregnant women seen at prenatal care program of a health organization	Einstein (Sao Paulo).	31826077	population (not c-section)
46	Moron AF.	2020	Infant microbiota and antibiotic prophylaxis in caesarean delivery	BJOG.	31628814	article type (not experimental)
47	Winther A, Axelsson PB, Clausen TD, Løkkegaard E.	2020	Prophylactic antibiotics in caesarean delivery before or after cord clamping - protecting the mother at the expense of the infant's microbiota?	BJOG.	31544335	article type (not experimental)
48	Tapiainen T, Koivusaari P, Brinkac L, Lorenzi HA, Salo J, Renko M, Pruikkonen H, Pokka T, Li W, Nelson K, Pirttilä AM, Tejesvi MV.	2019	Impact of intrapartum and postnatal antibiotics on the gut microbiome and emergence of antimicrobial resistance in infants	Sci Rep.	31337807	population (not mothers)

Supplement A.3 – Excluded Studies: Maternal Microbiome (Feles 2025)

#	Authors	Year	Title	Journal	PMID	Reason for exclusion
49	Neemann K, Olateju EK, Izevbigie N, Akaba G, Olanipekun GM, Richard JC, Duru CI, Kocmich NJ, Samson KK, Rezac-Elgohary A, Anigilaje EA, Yunusa T, Megafu CON, Ajose TO, Medugu N, Meza J, Obaro S.	2020	Neonatal outcomes associated with maternal recto-vaginal colonization with extended-spectrum β-lactamase producing Enterobacteriaceae in Nigeria: a prospective, cross-sectional study	Clin Microbiol Infect.	31336200	outcome (no (gut) microbiome analysis)
50	Zimmermann P, Curtis N.	2020	Effect of intrapartum antibiotics on the intestinal microbiota of infants: a systematic review	Arch Dis Child Fetal Neonatal Ed.	31296695	article type (not experimental)
51	Keskin M, Pabuccu EG, Sahin O, Cakmak D, Oral S, Kiseli M, Yarcı Gursoy A, Dincer Cengiz S.	2021	Oral antibiotic prophylaxis in elective cesarean deliveries: pilot analysis in tertiary Care Hospital	J Matern Fetal Neonatal Med.	31113268	outcome (no (gut) microbiome analysis)
52	Li H, Xiao B, Zhang Y, Xiao S, Luo J, Huang W.	2019	Impact of maternal intrapartum antibiotics on the initial oral microbiome of neonates	Pediatr Neonatol.	31056339	outcome (no (gut) microbiome analysis)
53	Kamal SS, Hyldig N, Krych Ł, Greisen G, Krogfelt KA, Zachariassen G, Nielsen DS.	2019	Impact of Early Exposure to Cefuroxime on the Composition of the Gut Microbiota in Infants Following Cesarean Delivery	J Pediatr.	31053348	population (not mothers)
54	Coker MO, Hoen AG, Dade E, Lundgren S, Li Z, Wong AD, Zens MS, Palys TJ, Morrison HG, Sogin ML, Baker ER, Karagas MR, Madan JC.	2020	Specific class of intrapartum antibiotics relates to maturation of the infant gut microbiota: a prospective cohort study	BJOG.	31006170	population (not mothers)
55	Merello M, Lotte L, Gonfrier S, Eleni Dit Trolli S, Casagrande F, Ruimy R, Bongain A.	2019	Enterobacteria vaginal colonization among patients with preterm premature rupture of membranes from 24 to 34 weeks of gestation and neonatal infection risk	J Gynecol Obstet Hum Reprod.	30562580	outcome (no (gut) microbiome analysis)
56	Committee on Practice Bulletins-Obstetrics.	2018	ACOG Practice Bulletin No. 199: Use of Prophylactic Antibiotics in Labor and Delivery	Obstet Gynecol.	30134425	article type (not experimental)
57	No authors listed	2018	ACOG Practice Bulletin No. 199 Summary: Use of Prophylactic Antibiotics in Labor and Delivery	Obstet Gynecol.	30134418	article type (not experimental)

Supplement A.3 – Excluded Studies: Maternal Microbiome (Feles 2025)

#	Authors	Year	Title	Journal	PMID	Reason for exclusion
58	Sood G, Argani C, Ghanem KG, Perl TM, Sheffield JS.	2018	Infections complicating cesarean delivery	Curr Opin Infect Dis.	29847329	article type (not experimental)
59	Stearns JC, Simioni J, Gunn E, McDonald H, Holloway AC, Thabane L, Mousseau A, Schertzer JD, Ratcliffe EM, Rossi L, Surette MG, Morrison KM, Hutton EK.	2017	Intrapartum antibiotics for GBS prophylaxis alter colonization patterns in the early infant gut microbiome of low risk infants	Sci Rep.	29184093	population (not mothers)
60	Nogacka A, Salazar N, Suárez M, Milani C, Arboleya S, Solís G, Fernández N, Alaez L, Hernández-Barranco AM, de Los Reyes-Gavilán CG, Ventura M, Gueimonde M.	2017	Impact of intrapartum antimicrobial prophylaxis upon the intestinal microbiota and the prevalence of antibiotic resistance genes in vaginally delivered full-term neonates	Microbiome.	28789705	population (not mothers)
61	Seedat F, Stinton C, Patterson J, Geppert J, Tan B, Robinson ER, McCarthy ND, Uthman OA, Freeman K, Johnson SA, Fraser H, Brown CS, Clarke A, Taylor-Phillips S.	2017	Adverse events in women and children who have received intrapartum antibiotic prophylaxis treatment: a systematic review	BMC Pregnancy Childbirth.	28747160	article type (not experimental)
62	Roesch LF, Silveira RC, Corso AL, Dobbler PT, Mai V, Rojas BS, Laureano ÁM, Procianoy RS.	2017	Diversity and composition of vaginal microbiota of pregnant women at risk for transmitting Group B Streptococcus treated with intrapartum penicillin	PLoS One.	28178310	outcome (no (gut) microbiome analysis)
63	Simioni J, Hutton EK, Gunn E, Holloway AC, Stearns JC, McDonald H, Mousseau A, Schertzer JD, Ratcliffe EM, Thabane L, Surette MG, Morrison KM.	2016	A comparison of intestinal microbiota in a population of low-risk infants exposed and not exposed to intrapartum antibiotics: The Baby & Microbiota of the Intestine cohort study protocol	BMC Pediatr.	27832763	other
64	Bookstaver PB, Bland CM, Griffin B, Stover KR, Eiland LS, McLaughlin M.	2015	A Review of Antibiotic Use in Pregnancy	Pharmacotherapy.	26598097	article type (not experimental)
65	Bailey SR, Field N, Townsend CL, Rodger AJ, Brocklehurst P.	2016	Antibiotic prophylaxis for women undergoing caesarean section and infant health	BJOG.	26435171	article type (not experimental)

Supplement A.3 – Excluded Studies: Maternal Microbiome (Feles 2025)

#	Authors	Year	Title	Journal	PMID	Reason for exclusion
66	Azad MB, Konya T, Persaud RR, Guttman DS, Chari RS, Field CJ, Sears MR, Mandhane PJ, Turvey SE, Subbarao P, Becker AB, Scott JA, Kozyrskyj AL	2016	Impact of maternal intrapartum antibiotics, method of birth and breastfeeding on gut microbiota during the first year of life: a prospective cohort study	BJOG.	26412384	population (not mothers)
67	Benito D, Lozano C, Jiménez E, Albújar M, Gómez A, Rodríguez JM, Torres C.	2015	Characterization of Staphylococcus aureus strains isolated from faeces of healthy neonates and potential mother-to-infant microbial transmission through breastfeeding	FEMS Microbiol Ecol.	25764567	outcome (no (gut) microbiome analysis)
68	Berardi A, Rossi C, Guidotti I, Vellani G, Lugli L, Bacchi Reggiani ML, Ferrari F, Facchinetti F, Ferrari F.	2014	Factors associated with intrapartum transmission of group B Streptococcus	Pediatr Infect Dis J.	25037035	outcome (no (gut) microbiome analysis)
69	Panda S, El khader I, Casellas F, López Vivancos J, García Cors M, Santiago A, Cuenca S, Guarner F, Manichanh C.	2014	Short-term effect of antibiotics on human gut microbiota	PLoS One.	24748167	population (not c-section)
70	Aloisio I, Mazzola G, Corvaglia LT, Tonti G, Faldella G, Biavati B, Di Gioia D.	2014	Influence of intrapartum antibiotic prophylaxis against group B Streptococcus on the early newborn gut composition and evaluation of the anti-Streptococcus activity of Bifidobacterium strains	Appl Microbiol Biotechnol.	24687755	population (not mothers)
71	Keski-Nisula L, Kyynäräinen HR, Kärkkäinen U, Karhukorpi J, Heinonen S, Pekkanen J.	2013	Maternal intrapartum antibiotics and decreased vertical transmission of Lactobacillus to neonates during birth	Acta Paediatr.	23398392	outcome (no (gut) microbiome analysis)
72	Sakata H.	2012	Evaluation of intrapartum antibiotic prophylaxis for the prevention of early-onset group B streptococcal infection	J Infect Chemother.	22614121	outcome (no (gut) microbiome analysis)
73	Jauréguy F, Carton M, Panel P, Foucaud P, Butel MJ, Doucet-Populaire F.	2004	Effects of intrapartum penicillin prophylaxis on intestinal bacterial colonization in infants	J Clin Microbiol.	15528713	population (not mothers)

Supplement A.3 – Excluded Studies: Maternal Microbiome (Feles 2025)

#	Authors	Year	Title	Journal	PMID	Reason for exclusion
74	Grönlund MM, Lehtonen OP, Eerola E, Kero P.	1999	Fecal microflora in healthy infants born by different methods of delivery: permanent changes in intestinal flora after cesarean delivery	J Pediatr Gastroenterol Nutr.	9890463	population (not mothers)
75	Mavromanolakis E, Maraki S, Samonis G, Tselentis Y, Cranidis A.	1997	Effect of norfloxacin, trimethoprim-sulfamethoxazole and nitrofurantoin on fecal flora of women with recurrent urinary tract infections	J Chemother.	9210003	population (not c-section)
76	Ernest JM, Givner LB.	1994	A prospective, randomized, placebo-controlled trial of penicillin in preterm premature rupture of membranes	Am J Obstet Gynecol.	8116706	outcome (no (gut) microbiome analysis)
77	Borderon JC, Bernard JC, Vergnaud R, Gold F, Soutoul JH, Laugier J.	1980	[Effect of antibiotic therapy in the mother on the colonization of the newborn by enterobacteriaceae (author's transl)]	Arch Fr Pediatr.	6903438	other (report not retrievable)
78	Flynn NM, Lawrence RM.	1979	Antimicrobial prophylaxis	Med Clin North Am.	119118	article type (not experimental)
79	Wong R, Gee CL, Ledger WJ.	1978	Prophylactic use of cefazolin in monitored obstetric patients undergoing cesarean section	Obstet Gynecol.	351488	outcome (no (gut) microbiome analysis)
80	Chodak GW, Plaut ME.	1977	Use of systemic antibiotics for prophylaxis in surgery: a critical review	Arch Surg.	320960	article type (not experimental)
81	Reyman M, van Houten MA, van Baarle D, Bosch AATM, Man WH, Chu MLJN, Arp K, Watson RL, Sanders EAM, Fuentes S, Bogaert D	2019	Impact of delivery mode-associated gut microbiota dynamics on health in the first year of life	Nat Commun.	31676793	population (not mothers)
82	Ismail MA , Nelson KE, Larson P, Moses VK	1990	Selective effect of cefoxitin prophylaxis on post-cesarean-section microbial flora	J Reprod Med.	2406439	outcome (no (gut) microbiome analysis)
83	Miller RD, Crichton D	1968	Ampicillin prophylaxis in caesarean section	J Obstet Gynaecol.	n.a.	outcome (no (gut) microbiome analysis)
