# Supplementary material for: Impact of Perioperative Antibiotic Prophylaxis in Caesarean Section on the Maternal Gut Microbiome: A Systematic Review
Source: J Clin Med. 2025 Jul 18;14(14):5104. doi: 10.3390/jcm14145104 (PMC12295807; doi:10.3390/jcm14145104)
Supplement: Supplementary file 1 [file jcm-14-05104-s001.zip › Supplementary File_S5_PRISMA-S_Checklist.pdf]

PRISMA-S Checklist – Maternal Microbiome (Feles 2025)

Section/topic	#	Checklist item	Location(s) Reported
**INFORMATION SOURCES AND METHODS**			
Database name	1	Name each individual database searched, stating the platform for each.	p.3, Materials and Methods (paragraph 2)
Multi-database searching	2	If databases were searched simultaneously on a single platform, state the name of the platform, listing all of the databases searched.	Not applicable – all databases searched individually
Study registries	3	List any study registries searched.	p.3, Materials and Methods (paragraph 2)
Online resources and browsing	4	Describe any online or print source purposefully searched or browsed (e.g., tables of contents, print conference proceedings, web sites), and how this was done.	p.3, Materials and Methods (paragraph 2)
Citation searching	5	Indicate whether cited references or citing references were examined, and describe any methods used for locating cited/citing references (e.g., browsing reference lists, using a citation index, setting up email alerts for references citing included studies).	p.3, Materials and Methods

			(paragraph 2); Figure 1
Contacts	6	Indicate whether additional studies or data were sought by contacting authors, experts, manufacturers, or others.	Not applicable – no additional data obtained through contacting authors
Other methods	7	Describe any additional information sources or search methods used.	Not applicable – no other sources or methods used
**SEARCH STRATEGIES**			
Full search strategies	8	Include the search strategies for each database and information source, copied and pasted exactly as run.	Supplement A.1
Limits and restrictions	9	Specify that no limits were used, or describe any limits or restrictions applied to a search (e.g., date or time period, language, study design) and provide justification for their use.	p.3, Materials and Methods (paragraph 2)
Search filters	10	Indicate whether published search filters were used (as originally designed or modified), and if so, cite the filter(s) used.	Not applicable – no search filters used

Prior work	11	Indicate when search strategies from other literature reviews were adapted or reused for a substantive part or all of the search, citing the previous review(s).	Not applicable – no strategies from other reviews were reused
Updates	12	Report the methods used to update the search(es) (e.g., rerunning searches, email alerts).	Not applicable – no search update conducted
Dates of searches	13	For each search strategy, provide the date when the last search occurred.	Supplement A.1
**PEER REVIEW**			
Peer review	14	Describe any search peer review process.	Not applicable – no formal search peer review conducted
**MANAGING RECORDS**			
Total Records	15	Document the total number of records identified from each database and other information sources.	Figure 1
Deduplication	16	Describe the processes and any software used to deduplicate records from multiple database searches and other information sources.	Figure 1 (duplicates removed manually using DOI/PubMed ID comparison in Excel)

PRISMA-S: An Extension to the PRISMA Statement for Reporting Literature Searches in Systematic Reviews
Rethlefsen ML, Kirtley S, Waffenschmidt S, Ayala AP, Moher D, Page MJ, Koffel JB, PRISMA-S Group.
Last updated February 27, 2020.
